# Antioxidant Defense and Redox Signaling in Elite Soccer Players: Insights into Muscle Function, Recovery, and Training Adaptations

**DOI:** 10.3390/antiox14070815

**Published:** 2025-07-02

**Authors:** Qing Meng, Chun-Hsien Su

**Affiliations:** 1 School of Physical Education, Huaqiao University, Xiamen 361021, China; mq@hqu.edu.cn; 2 Sport and Health Research Center, Huaqiao University, Xiamen 361021, China; 3 Department of Exercise and Health Promotion, Chinese Culture University, Taipei 111369, Taiwan; 4 College of Kinesiology and Health, Chinese Culture University, Taipei 111369, Taiwan

**Keywords:** mitochondrial biogenesis, exercise-induced stress, reactive species regulation, training periodization, injury prevention, biomarker monitoring

## Abstract

Elite soccer places significant neuromuscular and metabolic stress on athletes, leading to elevated production of reactive oxygen and nitrogen species (RONS), particularly in skeletal muscle, where intense contractile activity and increased oxygen flux drive oxidative processes. These reactive species play a dual role in skeletal muscle, supporting adaptive signaling at controlled levels while causing oxidative damage when poorly regulated. This paper presents an integrated synthesis of current knowledge on redox biology in elite soccer players, focusing on the origins and regulation of RONS, the functions of enzymatic and non-enzymatic antioxidant systems, and how both RONS and antioxidant responses influence muscle performance, fatigue, recovery, and long-term physiological adaptation. Drawing on studies conducted between 2000 and 2025, the discussion underscores the seasonal fluctuations in oxidative stress, individual variability in redox responses, and the potential adverse effects of unsystematic antioxidant supplementation. The analysis also emphasizes the value of using biomarker-guided, periodized antioxidant interventions tailored to training demands. Future directions include longitudinal tracking and the use of AI-assisted monitoring to enable personalized strategies for maintaining redox balance and optimizing performance in elite sport.

## 1. Introduction

Redox regulation is essential for muscle adaptation and performance in elite soccer, where players are regularly exposed to intense physical and metabolic stress. Reactive oxygen and nitrogen species (RONS), generated during exercise, influence muscle remodeling, fatigue, and recovery [1,2]. While moderate levels of RONS support physiological adaptation, excessive accumulation can disrupt cellular balance and contribute to oxidative stress and tissue damage [3,4]. The ability to maintain redox homeostasis through endogenous antioxidant systems is therefore critical to sustaining performance across training cycles and competitive demands [5].

This review integrates current knowledge on redox biology and antioxidant defense in elite soccer players. It begins with a foundational overview of the physiological stressors encountered in the sport and the key antioxidant systems in skeletal muscle. Subsequent sections explore molecular signaling mechanisms, redox biomarkers, and practical strategies for recovery and performance optimization.

### 1.1. Physiological Demands of Elite Soccer and Oxidative Load

The high-intensity, intermittent nature of elite soccer involves repeated sprints, rapid directional changes, jumping, and decelerative actions. These movement patterns result in elevated mitochondrial oxygen consumption and increased production of RONS in skeletal muscle, especially during eccentric contractions and transient ischemia–reperfusion events [6,7,8,9,10]. If not properly regulated, this oxidative load can impair calcium handling, promote inflammation, and delay muscle recovery [9,11].

The physiological strain is magnified during congested match schedules, where recovery periods are shortened and redox imbalances can accumulate [12,13]. Biomarkers such as creatine kinase and C-reactive protein have been shown to rise in response to such stress, reflecting muscle damage and systemic inflammation [10,14,15]. Managing oxidative stress through individualized recovery strategies, nutritional support, and training load adjustment is essential to prevent fatigue-related decline and reduce injury risk [11].

### 1.2. Antioxidant Systems in Skeletal Muscle

Skeletal muscle maintains redox balance through a coordinated antioxidant defense system. This includes enzymatic antioxidants such as superoxide dismutase (SOD), catalase (CAT), and glutathione peroxidase, which work to neutralize reactive species produced during exercise [9,10]. In addition, non-enzymatic antioxidants such as glutathione, vitamin C, and vitamin E support this system by stabilizing reactive intermediates and protecting cellular components from oxidative damage [16,17].

In elite athletes, regular training stimulates adaptations in the antioxidant system, improving the muscle’s ability to handle oxidative stress and enhancing recovery capacity [4,5]. However, these benefits can be diminished when antioxidant capacity is exceeded or when exogenous supplements are used inappropriately, potentially interfering with essential redox signaling pathways [18,19,20,21,22]. For this reason, antioxidant interventions should be carefully matched to the athlete’s training load and physiological condition in order to maintain adaptive benefits while avoiding disruption of beneficial molecular responses [9,23].

## 2. Literature Selection and Scope

This work utilizes a narrative approach to explore antioxidant defense mechanisms and redox signaling pathways in the context of elite soccer performance. The aim is to present an integrative perspective on skeletal muscle oxidative stress, adaptive responses, and their relevance to training, recovery, and performance optimization in high-level athletes. The discussion emphasizes conceptual synthesis and the practical application of redox biology to support effective performance management strategies.

The relevant literature was identified through a focused search of peer-reviewed journal articles published between 2000 and 2025 across databases including PubMed, Scopus, Web of Science, and EBSCO. Search terms were combinations of the following keywords: “reactive oxygen species”, “reactive nitrogen species”, “oxidative stress”, “antioxidants”, “skeletal muscle”, “soccer performance”, “redox signaling”, “exercise-induced adaptation”, and “training recovery”. Articles were selected based on their relevance to three core themes: (1) the physiological production and regulatory role of reactive species during exercise; (2) antioxidant defense systems in skeletal muscle; and (3) training and recovery strategies in elite soccer players that influence redox balance.

Both original research and review papers were considered to ensure comprehensive coverage of current knowledge. Studies involving human participants, exceptionally trained or elite athletic populations, were prioritized. Research with direct implications for soccer-specific performance, rather than general fitness or clinical populations, was emphasized. Rather than following a strict systematic review protocol, this narrative review allows for thematic exploration across key domains, including mitochondrial adaptations, antioxidant enzyme regulation, oxidative stress biomarkers, periodization, and nutritional interventions. This flexible approach facilitates a critical discussion of emerging evidence, identifies knowledge gaps, and highlights practical considerations for sports scientists and performance practitioners.

## 3. Redox Biology and Antioxidant Defenses in Skeletal Muscle

### 3.1. Sources and Types of Reactive Species During Exercise

During intense physical activity, particularly under the high-intensity, intermittent demands of elite soccer, the generation of reactive oxygen and nitrogen species (RONS) increases markedly. These include reactive oxygen species (ROS), such as superoxide anion (O_2_•^−^), hydrogen peroxide (H_2_O_2_), and hydroxyl radical (•OH), as well as reactive nitrogen species like nitric oxide (NO•) and its downstream product peroxynitrite (ONOO^−^), each varying in reactivity, half-life, and cellular target [24,25].

The mitochondria are the primary intracellular source of ROS during aerobic metabolism. Electron leakage at complexes I and III of the electron transport chain leads to partial reduction of oxygen and formation of superoxide, particularly under high ATP turnover in active muscle fibers [7]. Other enzymatic systems also contribute—NADPH oxidases (NOX2/NOX4) are activated by mechanical stress and inflammatory cues [26]; xanthine oxidase becomes active during ischemia–reperfusion events; and lipoxygenases and cyclooxygenases contribute additional ROS under inflammatory conditions [25].

A key component of RNS production is nitric oxide (NO), which plays an essential role in skeletal muscle vasodilation, mitochondrial respiration regulation, and redox signaling. NO is synthesized from L-arginine by the enzyme nitric oxide synthase (NOS), which exists in three isoforms: neuronal NOS (nNOS), endothelial NOS (eNOS), and inducible NOS (iNOS) [24]. In skeletal muscle, nNOS is the dominant isoform and is localized near the sarcolemma, where it regulates local blood flow, glucose uptake, and contractile efficiency [25]. eNOS, primarily expressed in vascular endothelium, contributes to perfusion regulation via shear-stress–mediated NO release [26]. Under physiological conditions, nNOS and eNOS generate NO in a controlled manner, supporting adaptive cellular processes.

However, under oxidative stress or cofactor depletion—such as reduced tetrahydrobiopterin availability—NOS may become uncoupled, producing superoxide instead of NO [27]. Furthermore, NO can rapidly react with superoxide to form peroxynitrite (ONOO^−^), a potent oxidant that can damage proteins, lipids, and nucleic acids. While NO plays a signaling role at physiological levels, its dysregulation contributes to nitrosative stress and impairs muscle function when homeostasis is lost [28].

The extent and location of RONS generation are influenced by contraction type, oxygen availability, and fiber type composition. Fast-twitch (type II) fibers, which dominate sprinting and explosive efforts, are particularly prone to oxidative stress due to high metabolic activity and relatively low antioxidant capacity [28,29]. These fibers are heavily recruited in elite soccer, especially during repeated efforts under fatigue.

In addition, cycles of restricted blood flow and reoxygenation—as seen during intermittent play—mimic ischemia–reperfusion conditions, further elevating ROS and RNS levels [30]. This dynamic redox environment reinforces the need for well-coordinated antioxidant defenses and recovery strategies tailored to the demands of elite soccer competition.

### 3.2. Antioxidant Defense Systems in Skeletal Muscle

To counteract exercise-induced RONS, skeletal muscle relies on a well-organized antioxidant defense system composed of enzymatic and non-enzymatic components. These systems maintain redox homeostasis, protect muscle integrity, and facilitate recovery and adaptation [31].

The primary enzymatic antioxidants include superoxide dismutase (SOD), CAT, and glutathione peroxidase (GPx). SOD converts superoxide into hydrogen peroxide, which is then broken down by CAT or reduced by GPx using glutathione as a substrate. These enzymes are compartmentalized within the cell: SOD1 is cytosolic; SOD2 is mitochondrial; and CAT resides in peroxisomes [31,32,33]. Their expression is regulated by redox-sensitive transcription factors, particularly Nuclear Factor Erythroid 2–Related Factor 2 (Nrf2), which is activated under oxidative stress to enhance antioxidant gene expression [31].

Complementing the enzymatic defenses are non-enzymatic antioxidants such as reduced glutathione (GSH), vitamin C, and vitamin E [34,35,36]. GSH is a direct scavenger and a cofactor in enzymatic detoxification [34]. Vitamin C exerts its antioxidant effects primarily in aqueous compartments, where it neutralizes free radicals and facilitates the regeneration of oxidized vitamin E to its active, reduced form, thereby preserving lipid membrane stability and extending the functional lifespan of vitamin E molecules [35]. In turn, vitamin E contributes to the stabilization of lipid membranes by preventing lipid peroxidation [37]. Additionally, dietary polyphenols such as quercetin, curcumin, and resveratrol play a role by modulating oxidative signaling pathways and reducing inflammation [38].

In elite athletes, these antioxidant systems are not static but dynamically adapt to training load, recovery status, and nutritional intake. As seen in training, regular exposure to moderate oxidative stress can upregulate these systems, enhancing resilience and performance [4]. However, disruptions in this balance, through overtraining, poor recovery, or inappropriate supplementation, can compromise adaptation [39]. Thus, understanding and supporting these endogenous defenses are critical for optimizing athletic output and long-term physiological health.

### 3.3. Dual Role of RONS: Signaling and Damage

Reactive oxygen and nitrogen species (RONS) exert a concentration-dependent influence on skeletal muscle, functioning as critical signaling molecules and potential sources of cellular damage. At physiological levels, RONS are indispensable for initiating and regulating adaptations to exercise [7]. They activate several redox-sensitive signaling pathways, including AMP-activated protein kinase (AMPK), mitogen-activated protein kinases (MAPKs), and Nrf2 [7,40]. These pathways mediate key processes such as mitochondrial biogenesis, antioxidant enzyme upregulation, glucose uptake, and muscle fiber remodeling [41,42].

Nrf2, in particular, serves as a central regulatory node in oxidative stress responses. Upon activation by moderate RONS levels, Nrf2 translocates to the nucleus [43]. It binds to antioxidant response elements (AREs), promoting the transcription of genes encoding detoxifying and antioxidant enzymes like SOD and GPx [43,44]. Similarly, AMPK and MAPKs are activated under increased oxidative load and energy stress, facilitating metabolic flexibility and enhancing cellular resilience [45]. Through these mechanisms, RONS are necessary stimuli that drive the adaptations required for improved muscle performance and recovery [43].

However, when the production of RONS exceeds the buffering capacity of the antioxidant defense system, oxidative stress occurs. This pathological condition is marked by structural and functional impairments. Elevated levels of RONS can trigger lipid peroxidation, protein carbonylation, and DNA damage, ultimately compromising cellular integrity and interfering with essential physiological functions [46]. In muscle cells, oxidative modifications to calcium-handling proteins, such as ryanodine receptors and Sarcoplasmic/Endoplasmic Reticulum Calcium ATPase (SERCA) pumps, interfere with excitation–contraction coupling [28,47]. This impairs calcium homeostasis, reducing contractile efficiency and delaying recovery [46].

Such oxidative disruptions are particularly detrimental in elite soccer players, who often face high-frequency neuromuscular demands and condensed recovery periods. Persistent oxidative stress diminishes short-term performance and contributes to chronic fatigue, muscle soreness, systemic inflammation, and elevated injury risk [2,48]. Importantly, this damage is not simply a byproduct of overexertion; it also reflects a failure of the redox system to adapt to cumulative training and match stress [49].

Therefore, maintaining a precise balance in RONS levels is critical enough to stimulate beneficial adaptations, but not so much as to trigger deleterious effects [50]. This delicate interplay emphasizes the need for targeted strategies that support redox homeostasis through well-regulated training loads, recovery protocols, and nutrition [48]. Recognizing the dual nature of RONS as both signal and stressor provides the foundation for developing individualized approaches to optimize performance while safeguarding long-term muscular health. A summarized comparison of reactive species sources, antioxidant defenses, and their dual role in muscle physiology is presented in Table 1.

## 4. Redox Homeostasis and Exercise in Elite Soccer Players

### 4.1. Exercise Demands and RONS Generation in Soccer

Elite soccer is defined by high-intensity, intermittent efforts that require a combination of aerobic endurance, anaerobic bursts, and rapid neuromuscular responses. Players frequently perform sprints, jumps, tackles, and directional changes, which place considerable metabolic and mechanical stress on the body. These repeated efforts elevate mitochondrial respiration, increasing electron leakage at complexes I and III of the electron transport chain and promoting superoxide formation [51,52]. This oxidative load intensifies as match intensity increases, with soccer matches often involving over 150 high-intensity actions [52].

In addition to mitochondrial sources, nonmitochondrial enzymes such as NADPH oxidase (NOX), xanthine oxidase, and nitric oxide synthase isoforms including nNOS and eNOS are activated in response to mechanical loading, inflammation, and transient hypoxia, all of which are frequently encountered during competitive play [53]. Eccentric muscle contractions, including those involved in braking, cutting, and deceleration, further increase RONS production by causing localized muscle damage and triggering inflammatory responses [54].

Additionally, the intermittent nature of play leads to repeated cycles of restricted blood flow and reoxygenation, mimicking ischemia–reperfusion conditions that favor oxidative reactions. These physiological stressors are exacerbated during congested match schedules, limiting recovery time and overwhelming antioxidant defenses [55]. If not properly regulated, the resulting oxidative imbalance may disrupt cellular signaling, impair calcium handling, and initiate catabolic processes. These effects can ultimately reduce athletic performance and increase the risk of injury [15].

Understanding the specific contexts in which RONS production is elevated, such as during sprints, directional changes, or periods of high match load, can inform the design of targeted training, recovery, and antioxidant strategies that preserve redox balance without suppressing necessary adaptive signals [56].

### 4.2. Acute and Chronic Oxidative Stress Responses

Acute bouts of high-intensity exercise transiently elevate RONS production, primarily in skeletal muscle. This short-term oxidative stress activates redox-sensitive signaling pathways such as AMP-activated protein kinase (AMPK), mitogen-activated protein kinases (MAPKs), and Nuclear Factor Erythroid 2–Related Factor 2 (Nrf2) [40,57]. These pathways promote mitochondrial biogenesis, antioxidant enzyme expression, and tissue repair mechanisms, supporting improved recovery and muscular resilience [40].

In contrast, chronic oxidative stress arises from repeated intense efforts without adequate recovery. Persistent RONS exposure may exceed the muscle’s adaptive capacity, leading to lipid peroxidation, protein carbonylation, DNA damage, and mitochondrial dysfunction [58]. In elite soccer players, this can impair calcium handling, neuromuscular function, and elevate inflammation, ultimately contributing to performance decline and increased injury risk [21].

These opposing outcomes reflect the principle of hormesis, where moderate oxidative stress promotes beneficial adaptations, while excessive stress is detrimental [59]. Well-trained athletes often exhibit enhanced antioxidant defenses, including increased expression of SOD, GPx, and CAT, and elevated levels of glutathione and vitamin C [4]. However, individual responses vary depending on genetic background, training load, nutrition, and recovery strategies.

**Figure 1:**
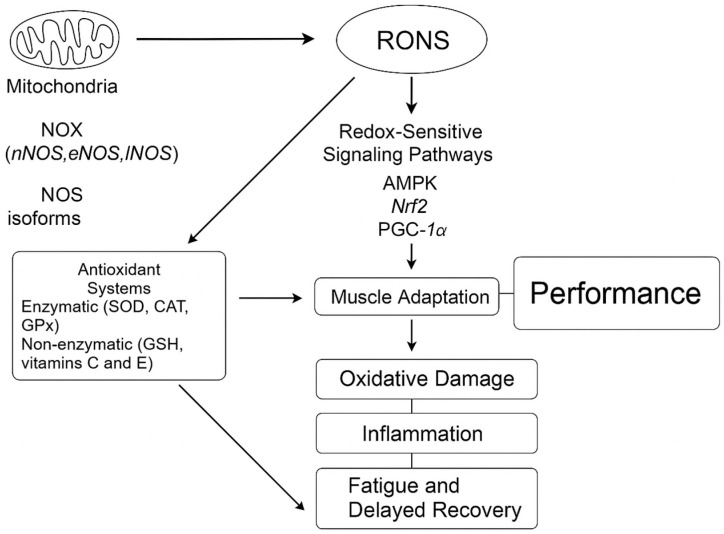
Redox regulation in elite soccer performance. During high-intensity exercise, reactive oxygen and nitrogen species (RONS) are produced by mitochondria, NADPH oxidases (NOX), and nitric oxide synthase (NOS) isoforms. Moderate levels of RONS activate redox-sensitive signaling pathways, such as AMPK, Nrf2, and PGC-1α, promoting muscle adaptation and performance. Antioxidant systems—including enzymatic (SOD, CAT, GPx) and non-enzymatic (GSH, vitamins C and E) components—counteract oxidative overload. Imbalance between RONS production and antioxidant capacity leads to oxidative damage, inflammation, and fatigue, ultimately impairing recovery and performance.

Given this variability, redox management in elite soccer cannot follow a one-size-fits-all approach. Instead, recovery and nutritional strategies should be personalized based on regularly monitoring redox biomarkers, training load, and individual responsiveness [3], thereby reducing the risk of oxidative maladaptation while promoting consistent performance. To better illustrate the complex interplay between RONS production, antioxidant defense, redox signaling, and athletic performance, a schematic overview is provided in Figure 1. This diagram integrates the molecular pathways discussed and highlights their implications for fatigue, recovery, and adaptation in elite soccer players.

### 4.3. Biomarker Monitoring and Practical Applications

Effectively managing redox homeostasis in elite athletes requires objective tools to assess oxidative stress and antioxidant status. Biomarkers offer a non-invasive and quantifiable way to monitor the balance between RONS production and antioxidant capacity, providing insights into an athlete’s physiological state and recovery needs.

Commonly used oxidative stress biomarkers include malondialdehyde (MDA) and F_2_-isoprostanes for lipid peroxidation, protein carbonyls for protein oxidation, and 8-hydroxy-2′-deoxyguanosine (8-OHdG) for oxidative DNA damage [60,61,62]. These can be measured through blood, urine, or saliva, making them practical for laboratory and field settings. Complementing these are markers of antioxidant defense, such as total antioxidant capacity (TAC) and activities of enzymes like SOD, CAT, and GPx.

When incorporated into athlete monitoring systems, these biomarkers help identify redox imbalance before it results in noticeable fatigue or performance drops [63,64]. This allows for proactive adjustments in training loads, recovery protocols, and dietary support. Advances in portable diagnostics have also improved the feasibility of frequent sampling, enabling real-time feedback [65].

It is important to interpret biomarker data within the appropriate physiological and training context. Isolated measurements may be misleading; therefore, longitudinal tracking that considers training phases, performance indicators, and subjective recovery assessments is essential for accurate evaluation [66]. Personalized profiles can guide evidence-based decisions on antioxidant use and recovery interventions, reducing injury risk and supporting long-term adaptation [64].

Redox biomarker monitoring, combined with other physiological and psychological indicators (e.g., HRV, neuromuscular function, perceived exertion), provides a comprehensive framework for optimizing training and recovery in elite soccer [63,67]. It marks a key step toward precision performance management tailored to the unique oxidative demands of each athlete. A synthesized overview of exercise-induced redox stress, adaptation mechanisms, and biomarker applications in elite soccer is presented in Table 2.

## 5. Redox Signaling and Muscle Function in Soccer Performance

### 5.1. Redox Regulation of Neuromuscular Function and Energy Metabolism

RONS exert significant control over neuromuscular function by modulating the molecular processes that govern excitation–contraction (E–C) coupling and cellular energy metabolism. In skeletal muscle, E–C coupling involves the precise sequence of electrical stimulation, calcium release, actin–myosin interaction, and subsequent calcium reuptake [68,69]. Moderate concentrations of RONS, particularly hydrogen peroxide (H_2_O_2_) and nitric oxide (NO•), can enhance this process by reversibly oxidizing thiol groups on proteins such as ryanodine receptors (RyR1) and SERCA pumps, thereby facilitating calcium handling and improving contractile performance [68,70].

This redox-sensitive modulation is especially relevant during submaximal or repetitive efforts common in elite soccer, where efficient calcium cycling supports sustained muscular output [71]. However, when RONS accumulate excessively, they induce irreversible oxidative modifications, such as carbonylation or nitrosylation, of contractile proteins, impairing calcium flux and reducing muscle fiber responsiveness [72,73]. This deterioration in E–C coupling contributes to decreased force production, particularly in later stages of match play or under cumulative fatigue [73].

In addition to regulating contractile function, RONS influence skeletal muscle energy metabolism by activating AMPK, a key energy sensor that responds to increased AMP: ATP ratios and oxidative cues [74]. Once activated, AMPK promotes adaptive processes including glucose uptake via GLUT4 translocation, fatty acid oxidation, and mitochondrial biogenesis [75]. These effects are primarily mediated through upregulation of peroxisome proliferator-activated receptor gamma coactivator 1-alpha (PGC-1α), a master regulator of oxidative metabolism and mitochondrial function [75].

The interaction between RONS, AMPK, and PGC-1α enhances metabolic flexibility, allowing soccer players to transition efficiently between energy systems during variable-intensity play [40]. Redox-sensitive pathways involving sirtuins (e.g., SIRT1) and FOXO transcription factors support mitochondrial quality control and protect against oxidative injury [41]. However, sustained oxidative overload can interfere with these signaling pathways, impair the mitochondrial membrane potential, and decrease ATP synthesis. These effects collectively compromise endurance capacity and contribute to increased fatigue.

Therefore, maintaining RONS within a functional range is crucial for preserving neuromuscular performance and metabolic resilience. Training, recovery, and nutritional strategies must be designed with an understanding of redox dynamics to support performance and adaptation without tipping into oxidative dysfunction.

### 5.2. Redox Contribution to Fatigue, Recovery, and Muscle Damage

In elite soccer, repeated high-intensity efforts with limited recovery elevate RONS production, contributing to fatigue and muscle stress [13,73]. While moderate RONS levels promote beneficial adaptations, excessive accumulation disrupts cellular functions essential for endurance and recovery [76]. A key mechanism involves impairment of excitation–contraction coupling through oxidative modification of calcium-handling proteins such as ryanodine receptor 1 (RyR1) and sarcoplasmic/endoplasmic reticulum Ca^2+^-ATPase (SERCA), leading to reduced calcium availability and slower reuptake [73]. Concurrently, oxidation of metabolic enzymes like glyceraldehyde-3-phosphate dehydrogenase and creatine kinase impairs ATP regeneration, compromising energy supply during prolonged activity [73].

These effects are pronounced in fast-twitch fibers, which are preferentially recruited during sprinting and possess lower antioxidant capacity [2]. Additionally, eccentric contractions cause structural muscle damage that initiates an inflammatory response, further amplifying RONS production through immune cell infiltration [77]. The resulting oxidative stress prolongs inflammation, delays repair processes, and contributes to delayed-onset muscle soreness (DOMS) and increased injury risk when recovery is inadequate [78].

Despite these risks, RONS are vital for initiating the repair and remodeling processes necessary for long-term adaptation [79]. They activate transcription factors such as NF-κB and AP-1, which upregulate cytokines and growth factors involved in muscle regeneration, satellite cell activation, and extracellular matrix remodeling [72]. Consequently, completely suppressing RONS, such as through indiscriminate antioxidant use, may interfere with natural recovery processes and hinder adaptation.

Effective recovery management, therefore, requires a nuanced understanding of redox physiology. Interventions to limit excessive oxidative stress (e.g., antioxidant support, cryotherapy) should be timed strategically to avoid blunting redox-sensitive adaptation pathways. By combining redox biomarker monitoring with neuromuscular assessments and subjective fatigue ratings, practitioners can better support muscle recovery and maintain high-level performance throughout the competitive season. A summary of redox signaling pathways and their influence on muscle function, fatigue, and recovery in elite soccer is presented in Table 3.

## 6. Antioxidant Defense and Recovery Strategies

### 6.1. Endogenous Adaptation Versus Exogenous Antioxidant Supplementation

Skeletal muscle has a well-developed endogenous antioxidant system that adjusts in response to oxidative stress induced by training. This adaptive response is governed by the principle of hormesis, in which exposure to moderate oxidative stress activates protective cellular mechanisms, thereby enhancing resilience and overall performance capacity. Central to this process is the transcription factor Nrf2, which, upon activation by RONS, translocates to the nucleus and promotes the expression of antioxidant enzymes such as SOD, CAT, and GPx, as well as enzymes involved in glutathione synthesis [2,80,81].

These adaptive responses enhance mitochondrial efficiency, reinforce redox buffering capacity, and assist in the regulation of inflammation. Together, these effects are essential for maintaining high-level athletic performance. Importantly, repeated exposure to manageable oxidative stress during training triggers the expression of PGC-1α, a key coactivator of mitochondrial biogenesis and oxidative metabolism [82]. This leads to improved endurance, delayed onset of fatigue, and greater efficiency in energy utilization, all of which are essential attributes for elite soccer players.

On the other hand, exogenous antioxidant supplementation introduces a layer of complexity. Widely used compounds such as vitamin C, E, and N-acetylcysteine (NAC) are intended to reduce oxidative damage and inflammation [22,83]. At the same time, polyphenols like quercetin, curcumin, and resveratrol have additional signaling effects [84]. While these supplements can reduce muscle soreness and improve subjective recovery in certain contexts, their indiscriminate or chronic use may interfere with the very signaling pathways that mediate beneficial training adaptations [84].

Studies have shown that high doses of antioxidants can blunt the activation of redox-sensitive kinases such as AMPK and p38 MAPK, reducing PGC-1α expression and impairing mitochondrial and metabolic adaptations [22,83,85]. These findings challenge the notion that oxidative stress is always harmful, highlighting its essential role in driving physiological improvements.

Thus, while antioxidant supplementation may be useful in specific contexts, such as injury recovery, high-altitude exposure, or during periods of extreme match congestion, it should not be applied universally. A periodized and personalized approach, aligned with training goals, recovery needs, and individual redox profiles, is increasingly regarded as the most effective strategy to optimize both adaptation and performance in elite soccer.

### 6.2. Nutritional and Physiological Recovery Interventions

In elite soccer, recovery strategies are crucial to counteract the neuromuscular and oxidative strain from frequent high-intensity activity. Both nutritional antioxidants and physiological methods are used to support tissue repair, reduce inflammation, and maintain performance continuity, though their effectiveness depends heavily on timing, context, and dosage.

From a nutritional perspective, several antioxidants are commonly used to buffer exercise-induced oxidative stress. Vitamin C operates in the aqueous phase to scavenge radicals and regenerate other antioxidants, while vitamin E protects lipid membranes from peroxidation [86]. NAC serves as a precursor for glutathione synthesis and has shown promise in preserving muscle function under high oxidative loads [87]. Polyphenols like quercetin, resveratrol, and curcumin offer dual benefits: direct antioxidant activity and modulation of redox-sensitive signaling pathways such as NF-κB and Nrf2 [88].

These compounds can be delivered through diet or supplements, but their absorption, efficacy, and interaction with training stress must be carefully considered. For instance, while acute supplementation may reduce inflammation and muscle damage post-exercise [89], chronic high-dose intake may suppress signaling pathways required for adaptation [90]. This underscores the need to align antioxidant intake with the training cycle, applying them more liberally during high-stress periods and sparingly during adaptation-focused blocks [26,91].

Physiological interventions such as cold-water immersion and cryotherapy are also widely used to manage inflammation and oxidative stress [92]. By limiting neutrophil activity and oxidative bursts following muscle damage, these techniques can aid short-term recovery. However, excessive or untimely use may blunt anabolic signaling and delay long-term adaptation [93].

Sleep is another vital component of redox regulation, as it influences hormonal rhythms and endogenous antioxidant activity, including melatonin production [94]. Sleep deprivation can impair redox signaling and reduce antioxidant capacity, emphasizing the need for consistent sleep hygiene as a non-negotiable element of recovery [95]. Additionally, low-intensity active recovery and massage can improve circulation, promote metabolic byproduct clearance, and reduce oxidative load [96].

Taken together, these interventions form a multifaceted recovery toolkit. Their success depends on individual responsiveness, the training phase, and the broader performance context. When strategically implemented, they can accelerate recovery, preserve adaptations, and extend the competitive longevity of elite soccer players.

### 6.3. Strategic Application and the Adaptation-Blunting Debate

Antioxidant strategies in elite sport must be applied with precision, as inappropriate timing or excessive dosing may impair training-induced adaptations. The adaptation-blunting hypothesis suggests that high-dose supplementation can suppress redox-sensitive pathways, such as AMPK, PGC-1α, and Nrf2, disrupting mitochondrial biogenesis and metabolic remodeling [26,97]. To preserve the benefits of oxidative signaling, antioxidant use should be aligned with specific training phases and individual recovery needs.

In elite soccer, where training focus shifts throughout the year, from capacity-building in the preseason to performance preservation during congested match periods, antioxidant strategies must be periodized [9]. During phases of high training load or competition density, moderate antioxidant use may help manage cumulative fatigue and protect against excessive oxidative stress [98]. However, during adaptation-intensive blocks, minimizing supplementation may preserve the oxidative signals necessary for physiological gains.

Individual variability further complicates blanket recommendations. Genetic polymorphisms in redox-related enzymes (e.g., SOD2, GSTP1), baseline antioxidant status, dietary habits, and positional demands all influence an athlete’s oxidative profile and responsiveness to interventions [1,99]. For example, midfielders and wide players typically engage in more high-intensity running than defenders or goalkeepers, potentially experiencing greater oxidative stress.

To tailor antioxidant support, objective data are essential. Biomarkers such as MDA, protein carbonyls, 8-OHdG, and enzyme activities (e.g., SOD, GPx) can provide insights into redox status when interpreted alongside performance and recovery metrics [26,100]. This enables practitioners to detect maladaptation early and adjust interventions accordingly.

Ultimately, the future application of antioxidants in elite sport is expected to focus on precision recovery. This approach involves the use of biomarker data, training load metrics, and athlete feedback to guide decisions regarding the timing, dosage, and method of intervention [101,102]. By doing so, it ensures that performance is supported without interfering with the molecular pathways essential for long-term development and physiological resilience. A comparative summary of antioxidant strategies, adaptive responses, and recovery interventions in elite soccer is presented in Table 4.

## 7. Periodization, Redox Adaptation, and Long-Term Conditioning

### 7.1. Seasonal Variation and Oxidative Stress Profiles

Throughout the annual training and competition cycle, elite soccer players are exposed to varying levels of physiological stress that impact their oxidative status. The different phases of the season, including the preseason, in-season, and off-season, each present unique metabolic, neuromuscular, and recovery demands that collectively influence redox balance [103,104]. Understanding how oxidative stress fluctuates across these periods is essential for designing effective training and recovery programs that support long-term conditioning and resilience.

During the preseason, training loads intensify as players rebuild fitness, strength, and coordination. This phase typically includes unfamiliar or high-volume eccentric exercises and increased total workload, all of which elevate RONS production [2,80]. Mitochondrial respiration, NADPH oxidase activity, and inflammatory responses from muscle microtrauma contribute to elevated oxidative stress, as reflected by increased biomarkers such as MDA and protein carbonyls [105]. However, when managed properly, this oxidative challenge acts as a beneficial stimulus, activating redox-sensitive pathways like Nrf2 and promoting antioxidant enzyme expression and mitochondrial biogenesis [106]. These adaptations lay the physiological foundation for enduring performance throughout the season.

The in-season phase for elite soccer players is characterized by a focus on maintaining performance, preventing injuries, and ensuring efficient recovery between matches. This period is marked by frequent high-intensity games, travel, and limited rest, which collectively contribute to increased oxidative stress. For players with high playing minutes and limited rotation, the endogenous antioxidant capacity may be insufficient to counteract the repeated exposure to RONS. This persistent oxidative overload can lead to reduced recovery quality, elevated muscle damage markers, and a higher incidence of soft tissue injuries. Therefore, in-season management often requires targeted nutritional support, individualized recovery interventions, and close monitoring of oxidative stress to prevent maladaptation. Effective nutritional strategies for post-match recovery emphasize the timely intake of approximately 1.2 g/kg/h of carbohydrates and 40 g of protein within 20 min to restore glycogen and stimulate muscle protein synthesis. Daily recommendations include 6–10 g/kg of carbohydrates and over 1.5 g/kg of protein, ideally in divided doses [107]. Supplementation with vitamin D, omega-3 fatty acids, creatine, and antioxidants may further support recovery, with compounds like curcumin and bromelain showing potential benefits pending further research [108]. Monitoring oxidative stress through markers such as creatine kinase (CK) and C-reactive protein helps assess muscle damage and inflammation, as CK typically peaks 24–48 h post-match and normalizes within 72 h [9,14]. Antioxidant enzymes, including glutathione reductase and CAT, exhibit seasonal variation, suggesting adaptive buffering of oxidative stress, despite stable overall marker levels [2].

The off-season is a critical phase for redox recalibration in elite soccer players, providing an opportunity for physiological regeneration, reduction in systemic inflammation, and restoration of antioxidant capacity. Decreased training volume lowers oxidative stress, allowing replenishment of key antioxidants such as glutathione and vitamins C and E [26,109,110]. This period also supports improved sleep and circadian rhythm regulation, which enhances recovery and immune function [111,112]. Regular in-season oxidative stress may enhance endogenous defenses, which can be recalibrated during the off-season to restore redox balance [4]. Although antioxidant supplementation may aid recovery, its effectiveness depends on exercise intensity and individual requirements [26,110]. Low-level conditioning should be maintained to preserve mitochondrial function and prevent detraining [4,113], while the shift toward a more reducing environment supports long-term performance and health [113]. This phase also allows for dietary reassessment to better prepare for oxidative demands in the upcoming preseason [111,114].

By recognizing the seasonal rhythm of oxidative stress, practitioners can better align antioxidant strategies with physiological demands, ensuring recovery and adaptation are both supported throughout the training year.

### 7.2. Periodized Antioxidant Strategies and Individual Redox Profiling

Antioxidant strategies in elite soccer should be periodized to match the oxidative demands of specific training phases. High-dose supplementation during adaptation-focused periods, such as preseason or strength blocks, may blunt training-induced adaptations by interfering with redox-sensitive pathways like AMPK, PGC-1α, and Nrf2, which are essential for mitochondrial biogenesis and metabolic remodeling [97,115]. Endogenous antioxidant systems, strengthened through controlled oxidative stress, play a key role in promoting adaptation and performance by facilitating ROS-mediated signaling and reducing inflammation [26]. In contrast, during high-intensity competition phases, short-term antioxidant support may help mitigate fatigue and muscle damage, particularly when appropriately timed and dosed [26,116]. Although some evidence suggests that antioxidants can attenuate mitochondrial and antioxidant adaptations, the redundancy in skeletal muscle signaling pathways indicates that training may offset these effects in certain contexts [97,115].

In elite soccer players, individualized redox management is essential due to variability in oxidative stress susceptibility, influenced by genetic polymorphisms in antioxidant enzymes such as SOD2, GPX1, and CAT [50,117]. These genetic differences, along with training history, position-specific demands, and dietary patterns, affect oxidative stress responses and recovery needs [2,4,118]. Personalized nutritional interventions, including vitamin C and antioxidant-rich diets, can support redox balance and reduce markers like 8-OHdG and F2α-isoprostane [117,118]. Biomarker profiling, which includes measures such as MDA, 8-OHdG, protein carbonyls, and total antioxidant capacity, along with data from GPS tracking, HRV, and subjective fatigue assessments, provides the basis for developing individualized antioxidant strategies [118,119]. Applying these interventions across training cycles helps regulate oxidative stress, enhances performance, and reduces the risk of overtraining [4,119]. A seasonal and personalized overview of redox adaptation and antioxidant strategy in elite soccer is presented in Table 5.

## 8. Future Research Directions

Future investigations should prioritize longitudinal studies that capture redox fluctuations across an entire competitive season in elite soccer players. Current research predominantly focuses on acute responses to isolated training or matches, offering limited insight into cumulative oxidative stress and its implications for injury risk, fatigue, and adaptation. Monitoring oxidative biomarkers alongside performance and recovery metrics during preseason, in-season, and off-season phases would provide a more integrated understanding of redox dynamics and their role in athlete resilience and performance sustainability.

Advancements in omics technologies, including metabolomics and transcriptomics, provide new opportunities to explore redox-sensitive pathways involved in muscle adaptation. These approaches can assist in detecting early molecular changes that are associated with maladaptation or overtraining. Additionally, future studies should aim to develop individualized antioxidant strategies based on athletes’ genetic profiles, training demands, and biomarker data. Integrating these insights into AI-assisted monitoring systems could enable real-time decision-making in training and recovery planning, facilitating precision-based redox management tailored to each athlete’s physiological needs.

## 9. Conclusions

RONS play a central role in skeletal muscle physiology, particularly under the physical demands of elite soccer. At moderate levels, RONS serve as important signaling molecules that stimulate beneficial adaptations. These include mitochondrial biogenesis, induction of endogenous antioxidant enzymes, and muscle remodeling processes that collectively enhance endurance, recovery, and overall performance.

However, when RONS production exceeds the buffering capacity of antioxidant defenses due to excessive training loads, poor recovery, or suboptimal nutrition, the result can be maladaptive. Excess RONS may disrupt redox homeostasis, impair calcium signaling, damage mitochondrial function, and accelerate muscle fatigue and inflammation. Over time, this can lead to performance decline and a greater risk of soft tissue injuries.

Recognizing this dual nature of RONS, future strategies should avoid uniform antioxidant use and instead prioritize personalized, periodized approaches. These may include monitoring redox biomarkers, adapting antioxidant support to the athlete’s physiological state, and integrating advancements in digital performance tracking and nutrition science. Such individualized interventions can help maintain a balance that supports adaptive signaling while preventing oxidative damage, thereby promoting long-term athletic resilience and peak performance in elite soccer players.

## Figures and Tables

**Table 1:** Integrated overview of redox species, antioxidant defenses, and physiological effects in skeletal muscle during exercise.

Mechanism	Primary Sources and Examples	Key Molecular Targets/ Components	Effects at Physiological Levels	Effects when Excessive	Practical Implications for Elite Soccer Players	References
Reactive Species Generation	Mitochondria (Complex I and III), NADPH oxidase (NOX2/4), xanthine oxidase, uncoupled NOS	Superoxide (O_2_•^−^), H_2_O_2_, •OH, NO•, ONOO^−^	Signal transduction, mitochondrial biogenesis, and glucose uptake	Oxidative stress, lipid/protein/DNA damage, impaired calcium handling	Requires modulation through recovery protocols and load management	[7,24,25,26,27,28,30]
Antioxidant Defense—Enzymatic	SOD (cytosolic/mitochondrial), CAT, GPx	Detoxification of superoxide and hydrogen peroxide	Maintains redox homeostasis, supports adaptation	Inadequate during overtraining or insufficient recovery	Endogenous enzyme expression can be enhanced through training hormesis	[31,32,33,43]
Antioxidant Defense—Non-Enzymatic	Glutathione (GSH), vitamins C and E, polyphenols (quercetin, resveratrol)	Neutralization of free radicals, regeneration of enzymatic antioxidants	Protects membranes and proteins, supports recovery	Risk of adaptation blunting if over-supplemented	Timing and dosing of supplements must align with a training phase	[34,35,36,37,38]
Signaling Pathways	RONS-mediated activation of AMPK, Nrf2, MAPKs	Antioxidant gene transcription, mitochondrial biogenesis	Promotes metabolic flexibility, muscle remodeling	Suppressed adaptation if RONS is excessively neutralized	Controlled RONS exposure is essential for long-term conditioning	[7,40,41,42,43,44,45]
Muscle Fiber Susceptibility	Fast-twitch (Type II) fibers in sprinting, cutting	Lower intrinsic antioxidant levels	Greater capacity for power but higher vulnerability to oxidative damage	Increased injury risk, especially under congested schedules	Customized antioxidant support is needed based on positional demands	[28,29,49]

Note: for the full terms of abbreviations used in this table, please refer to the Abbreviations Section at the end of the manuscript.

**Table 2:** Overview of redox stress, adaptive responses, and biomarker-guided strategies in elite soccer.

Mechanism or Concept	Description and Key Features	Effects/Outcomes	Implications for Elite Soccer Players	References
RONS Generation in Match Play	High-intensity actions, ischemia–reperfusion cycles, eccentric contractions	Elevated oxidative load, disrupted calcium handling, and inflammation	Needs monitoring during congested schedules; impacts recovery and injury risk	[15,51,52,53,54,55]
Acute Oxidative Stress	Transient RONS production post-exercise; activates signaling pathways (e.g., AMPK, Nrf2)	Stimulates mitochondrial biogenesis, antioxidant enzyme expression	Essential for adaptation; should not be overly suppressed	[40,57]
Chronic Oxidative Stress	Repeated exposure due to overtraining, poor nutrition, and insufficient rest	Lipid/protein/DNA damage, mitochondrial dysfunction, fatigue	Leads to maladaptation and injury risk if unmanaged	[21,58]
Hormesis Principle	Moderate stress promotes adaptation; excessive stress causes damage	Enhances redox resilience vs. elevates injury and inflammation	Guides training load and antioxidant strategy design	[4,59]
Individual Variability	Influenced by genetics, age, training, and nutrition	Determines antioxidant capacity and stress response	Necessitates personalized recovery and supplementation plans	[3,4,21]
Oxidative Stress Biomarkers	MDA, 8-OHdG, F_2_-isoprostanes, protein carbonyls	Reflect lipid, DNA, and protein oxidation status	Enables early detection of redox imbalance	[60,61,62]
Antioxidant Defense Biomarkers	Total antioxidant capacity, SOD, CAT, GPx activity	Assess redox buffering capacity and recovery state	Support real-time monitoring and intervention tailoring	[63,64,65]
Practical Monitoring Applications	Longitudinal biomarker tracking + HRV, RPE, performance data	Informs precision training, recovery, and nutrition	Enhances resilience and minimizes maladaptation risks	[63,66,67]

**Table 3:** Summary of redox signaling effects on neuromuscular function, fatigue, and muscle recovery in elite soccer.

Mechanism or Concept	Description and Key Features	Effects/Outcomes	Implications for Elite Soccer Players	References
Excitation–Contraction (E–C) Coupling Modulation	RONS modulate RyR1 and SERCA through reversible oxidation	Enhances calcium handling and contractile performance	Supports sustained submaximal output; vulnerable to oxidative damage in fatigue	[68,69,70,71]
Excessive RONS and E–C Disruption	Irreversible protein oxidation impairs calcium flux	Reduces force production and muscle responsiveness	Leads to late-match fatigue, especially under high load	[72,73]
AMPK–PGC-1α Pathway Activation	AMPK is activated by oxidative and energetic stress	Promotes glucose uptake, FAO, mitochondrial biogenesis	Enhances energy efficiency and endurance performance	[40,74,75]
Redox and Mitochondrial Regulation	RONS-sensitive transcription (e.g., SIRT1, FOXO) affects mitochondrial health	Improves metabolic flexibility and quality control	Disrupted under chronic oxidative overload, reducing ATP	[40,41]
Fast-Twitch Fiber Susceptibility	Type II fibers with low antioxidant capacity under high load	Accumulate oxidative damage, reduce contraction efficiency	Require targeted recovery and antioxidant strategies	[2,73]
RONS Role in Muscle Damage and DOMS	Secondary oxidative stress from immune activation post-exercise	Lipid and protein oxidation, prolonged inflammation	Increases soreness and injury risk under poor recovery	[13,73,77,78]
RONS in Muscle Repair and Remodeling	Activate NF-κB and AP-1, stimulating cytokines and growth factors	Support satellite cell activation and muscle regeneration	Necessary for adaptation; indiscriminate antioxidant use may impair the process	[72,79]
Recovery Management Strategy	Combines redox biomarker monitoring with neuromuscular and fatigue data	Enables precise intervention timing (e.g., antioxidant, cryotherapy)	Prevents maladaptation and maintains performance	[2,76,79]

**Table 4:** Summary of endogenous and exogenous antioxidant strategies and recovery applications in elite soccer.

Mechanism or Concept	Description and Key Features	Effects/Outcomes	Implications for Elite Soccer Players	References
Endogenous Antioxidant Adaptation	Nrf2 activation induces antioxidant enzymes (SOD, CAT, GPx) in response to RONS	Enhances redox resilience and mitochondrial efficiency	Promotes natural adaptation; training must provide a moderate oxidative stimulus	[2,80,81]
PGC–1α–Mediated Adaptation	Triggered by oxidative and energetic stress; supports mitochondrial biogenesis and metabolism	Increases endurance, delays fatigue, and improves energy utilization	Critical for long-term adaptation and performance enhancement	[82]
Exogenous Supplementation—Vitamins C, E, NAC	Reduces oxidative stress and inflammation but may blunt signaling if misused	Can impair AMPK and PGC-1α activation, reducing adaptation	Use cautiously during injury or overload periods, not chronically	[22,83,85]
Polyphenol-Based Supplementation	Quercetin, curcumin, resveratrol have antioxidant and signaling effects	Aid inflammation control and recovery; modulate NF-κB and Nrf2	Apply selectively based on training intensity and goals	[84,88,90]
Physiological Interventions	Cryotherapy, cold-water immersion, sleep, massage, and active recovery	Support acute recovery and tissue repair; reduce inflammatory oxidative bursts	Effective when timed appropriately; avoid overuse to preserve adaptations	[92,93,94,95,96]
Adaptation-Blunting Hypothesis	High antioxidant doses suppress redox-sensitive pathways (AMPK, PGC-1α, Nrf2)	May limit mitochondrial biogenesis and oxidative capacity	Highlights the importance of precise timing and dosing	[26,83,97]
Training Periodization of Antioxidants	Strategy varies between adaptation-focused and performance-focused phases	Balances oxidative signaling and damage control	Periodized planning enhances recovery and preserves adaptation	[9,98]
Individual Variability and Precision Recovery	Redox response is influenced by genetics, diet, training, and playing position	Biomarkers (MDA, 8-OHdG, GPx) enable targeted intervention	Supports personalized antioxidant and recovery protocols	[1,26,99,100,101,102]

**Table 5:** Seasonal variation, periodized antioxidant strategy, and personalized redox profiling in elite soccer.

Mechanism or Concept	Description and Key Features	Effects/Outcomes	Implications for Elite Soccer Players	References
Preseason Redox Challenge	High training volume and eccentric exercises increase RONS via mitochondrial respiration, NOX activity, and inflammation	Activates Nrf2, enhances antioxidant enzyme expression, and promotes mitochondrial biogenesis	Forms an adaptive foundation for in-season performance; must be monitored for overload	[2,80,103,104,105,106]
In-Season Oxidative Load	Frequent high-intensity matches, travel, and limited rest elevate oxidative stress, CK, and inflammation	Can impair recovery, increase muscle damage, and raise injury risk	Demands targeted nutrition (CHO, protein, vitamin D), biomarker monitoring, and rotation strategies	[9,14,107,108]
Off-Season Redox Recalibration	Reduced load allows antioxidant restoration and inflammation resolution; improved sleep supports redox reset	Enhances glutathione, vitamin C/E levels, immune function, and mitochondrial maintenance	Ideal for physiological regeneration and dietary reassessment	[4,26,109,110,111,112,113,114]
Periodized Antioxidant Strategy	High-dose antioxidants during adaptation phases may suppress AMPK, PGC-1α, and Nrf2 pathways	May blunt training adaptations and mitochondrial gains	Strategy should match training cycle—minimal during adaptation blocks, moderate during congestion	[26,97,115,116]
Redox Genetic Variability	Individual differences in antioxidant enzyme genes (SOD2, GPX1, CAT) modulate oxidative response	Affects susceptibility to oxidative stress and efficacy of supplementation	Highlights the need for genetically informed nutrition and recovery protocols	[2,4,50,117]
Biomarker-Guided Personalization	Redox markers (MDA, 8-OHdG, protein carbonyls, TAC) with GPS, HRV, and RPE data provide a recovery profile	Supports individualized training, antioxidant timing, and stress management	Reduces overtraining risk and optimizes performance through precision conditioning	[4,117,118,119]

## Data Availability

Not applicable.

## Abbreviations

The following abbreviations are used in this manuscript:

RONS	Reactive Oxygen and Nitrogen Species
E–C coupling	Excitation–Contraction Coupling
ROS	Reactive Oxygen Species
NADPH	Nicotinamide Adenine Dinucleotide Phosphate
NOX	NADPH Oxidase
NOS	Nitric Oxide Synthase
SOD	Superoxide Dismutase
GPx	Glutathione Peroxidase
CAT	Catalase
GSH	Reduced Glutathione
AMPK	AMP-Activated Protein Kinase
PGC-1α	Peroxisome Proliferator-Activated Receptor Gamma Coactivator 1-alpha
Nrf2	Nuclear Factor Erythroid 2–Related Factor 2
NF-κB	Nuclear Factor Kappa-Light-Chain-Enhancer of Activated B Cells
TAC	Total Antioxidant Capacity
8-OHdG	8-Hydroxy-2′-Deoxyguanosine
DOMS	Delayed-Onset Muscle Soreness
CK	Creatine Kinase
HRV	Heart Rate Variability
SERCA	Sarcoplasmic/Endoplasmic Reticulum Calcium ATPase
MAPK	Mitogen-Activated Protein Kinase
FOXO	Forkhead Box O Transcription Factor
SIRT1	Sirtuin 1
CHO	Carbohydrates
NAC	N-Acetylcysteine
FAO	Fatty Acid Oxidation
GPS	Global Positioning System (Athlete Tracking)
RPE	Rating of Perceived Exertion
